# Identification and validation of diagnostic and prognostic biomarkers in prostate cancer based on WGCNA

**DOI:** 10.1007/s12672-024-00983-5

**Published:** 2024-09-21

**Authors:** Xi Xiao, Liangliang Qing, Zonglin Li, Fuxiang Ye, Yajia Dong, Jun Mi, Junqiang Tian

**Affiliations:** Department of Urology, Lanzhou University Second Hospital, Lanzhou, 730030 China

**Keywords:** Prostate cancer, Diagnosis, Prognosis, Biomarker, WGCNA

## Abstract

**Background:**

Prostate cancer (PCa) represents a significant health challenge for men, and the advancement of the disease often results in a grave prognosis for patients. Therefore, the identification of biomarkers associated with the diagnosis and prognosis of PCa holds paramount importance in patient health management.

**Methods:**

The datasets pertaining to PCa were retrieved from the Gene Expression Omnibus (GEO) database. Weighted gene co-expression network analysis (WGCNA) was conducted to investigate the modules specifically associated with the diagnosis of PCa. The hub genes were identified using the LASSO regression analysis. The expression levels of these hub genes were further validated by qRT-PCR experiments. Receiver operating characteristic (ROC) curves and nomograms were employed as evaluative measures for assessing the diagnostic value.

**Results:**

The blue module identified by WGCNA exhibited a strong association with PCa. Six hub genes (SLC14A1, COL4A6, MYOF, FLRT3, KRT15, and LAMB3) were identified by LASSO regression analysis. Further verification confirmed that these six genes were significantly downregulated in tumor tissues and cells. The six hub genes and the nomogram demonstrated substantial diagnostic value, with area under the curve (AUC) values ranging from 0.754 to 0.961. Moreover, patients with low expression levels of these six genes exhibited elevated T/N pathological stage and Gleason score, implying a more advanced disease state. Meanwhile, their progression-free survival (PFS) was observed to be potentially poorer. Finally, a significant association could be observed between the expression of these genes and the dysregulation of immune cells, along with drug sensitivity.

**Conclusions:**

In summary, our study identified six hub genes, namely SLC14A1, COL4A6, MYOF, FLRT3, KRT15, and LAMB3, which can be utilized to establish a diagnostic model for PCa. The discovery may offer potential molecular targets for clinical diagnosis and treatment of PCa.

**Supplementary Information:**

The online version contains supplementary material available at 10.1007/s12672-024-00983-5.

## Introduction

Prostate cancer (PCa) is the second most prevalent form of cancer among men worldwide and ranks fifth in terms of cancer-related mortality, imposing a significant healthcare burden on men [1]. It is widely recognized that the occurrence and progression of PCa involve multiple factors and processes, constituting a complex pathological transformation. However, the specific underlying mechanisms remain incompletely understood [2]. At present, the detection of prostate-specific antigen (PSA) plays a pivotal role in the early diagnosis of PCa, serving as a widely employed biomarker [3]. However, the diagnostic specificity of PSA for PCa is limited due to its association with benign prostatic hyperplasia, or prostatitis. Furthermore, a substantial proportion of PCa patients do not present with elevated serum PSA levels [4]. The occurrence of unnecessary biopsies and overtreatment based on PSA is also on the rise in clinical practice [5]. Simultaneously, there is currently no ideal tumor marker or molecular target that serves as the definitive evaluation basis for the diagnosis and individualized treatment of PCa [6]. Hence, the exploration of effective diagnostic biomarkers and therapeutic targets for PCa assumes paramount importance in aiding clinicians to make more precise decisions and enhancing patient prognosis.

With the rapid development of high-throughput sequencing technology, the scientific community has increasingly applied it to solve biological problems [7]. In medicine, high-throughput sequencing is widely used to identify candidate genes related to disease diagnosis, treatment, and prognosis. Weighted gene co-expression network analysis (WGCNA) can integrate highly related genes into the same module, thereby discovering the relationship between gene networks and phenotypes of interest, and identifying core genes in the network [8]. The WGCNA is very helpful in identifying biomarkers relevant to disease diagnosis and treatment and is commonly used in tumor-related studies, including liver cancer [9], bladder cancer [10], and Gastric cancer [11]. However, there are fewer studies on the application of WGCNA to search for PCa biomarkers, especially those that inhibit the development of PCa. In PCa, the downregulation of tumor suppressor genes plays an important role, exerting significant influence on key biological processes including cell growth, cell cycle regulation, DNA repair, and apoptosis. The downregulation of these genes may lead to the abnormal proliferation of tumor cells and their ability to evade normal regulation by the body [12, 13]. Therefore, a thorough investigation of these genes can enhance our understanding of the molecular mechanisms of PCa and contribute to the improvement of PCa diagnosis and treatment.

The aim of this study is to conduct a comprehensive analysis of PCa-related high-throughput datasets from the GEO database, and to identify biomarkers associated with PCa diagnosis and treatment using a machine learning approach. Additionally, we will validate these biomarkers using the PCa cohort in the TCGA database, assess their prognostic value and potential functions, and validate them at the expression level using specific cellular assays. Through these efforts, our study will provide significant insights into the diagnosis and management of PCa.

## Materials and methods

### Data collection and acquisition

We gathered data from 5 microarray datasets of PCa (GSE88808, GSE69223, GSE46602, GSE32571, and GSE32448) available in the GEO database. In these datasets, we obtained 199 PCa samples and 157 normal prostate samples. Subsequently, we applied the sva package [14] and the limma package [15] to standardize and correct the data. Additionally, we obtained gene expression data and corresponding clinical information of PCa from the TCGA database. In the TCGA database, there were a total of 501 tumor samples and 52 normal samples. Patients with missing clinical information were excluded from the clinical prognosis analysis. Detailed information on all cohorts can be found in Supplementary Table 1. Finally, we obtained immunohistochemical images of hub genes from the Human Protein Atlas (HPA) (https://www.proteinatlas.org/).

### Differential analysis

We utilized the “limma” package [15] to conduct an analysis of gene expression differences between PCa tissue and normal prostate tissue, using normalized data from the GEO cohort. To identify significant DEGs, the screening criteria for DEGs was |log2FC|> 1, p < 0.05. Finally, for data visualization, we employed the “pheatmap” [16] and “ggplot2” [17] packages to generate graphical representations of the results.

### Acquisition and validation of biomarkers for disease diagnosis

First, we utilized the “WGCNA” package [8] to perform WGCNA on the normalized GEO data. Based on the scale-free topology criterion, the optimal soft threshold in our analysis is determined to be 5. Subsequently, we employed hierarchical clustering analysis to identify distinct gene modules, setting the criterion for module size to be greater than 60. Next, we assessed the correlation between each module and PCa occurrence using Pearson correlation analysis. Subsequent to the identification of the most pertinent modules related to disease characteristics, the core genes within these modules were screened based on the criteria of having Module Membership (MM) greater than 0.8 and Gene Significance (GS) greater than 0.6. Furthermore, we identified the intersection genes of the previously identified DEGs with these core genes and performed Least Absolute Shrinkage and Selection Operator (LASSO) regression to further refine and select optimal features, thereby identifying the hub genes that can serve as biomarkers for disease diagnosis. Meanwhile, the expression of these genes was analyzed in the aforementioned GEO cohort, with further validation of their expression patterns achieved through data obtained from the TCGA database. Ultimately, we assessed the performance of these genes in predicting PCa occurrence by calculating the Area Under the Curve (AUC) using Receiver Operating Characteristic (ROC) curve analysis with the “pROC” package [18].

### Construction and verification of disease prediction nomogram

To enhance the accuracy of disease prediction, we employed logistic regression to establish a nomogram incorporating the hub genes identified in our analysis. Subsequently, we validated the performance of the nomogram using various statistical methods, including ROC curve analysis, calibration curve analysis [19], Decision Curve Analysis (DCA) [20], and clinical impact curve analysis.

### Immune correlation analysis

We conducted a single-sample gene set enrichment analysis (ssGSEA) [19] utilizing the “GSVA” package to assess the infiltration of 28 immune cell types in samples of PCa and normal prostate tissue [21]. The obtained results were visually represented using heatmaps and violin plots generated with the aid of the “pheatmap” [16] and “vioplot” [22] packages, respectively. Subsequently, the association between the hub genes and the infiltration of immune cells was assessed through the application of Spearman's rank correlation method [23].

### Functional analysis and drug sensitivity analysis

The “clusterProfiler” package was employed for performing Gene Set Enrichment Analysis (GSEA) [24] for each hub gene in the PCa cohort, with hallmark gene sets selected as the enriched gene sets. For drug sensitivity analysis, we utilized the “oncoPredict” package [25], which is designed for predicting in vivo drug responses in cancer patients. This analysis involved 198 drugs, and we filtered drugs that were associated with these hub genes based on a significance threshold of p < 0.05.

### Clinical characteristics and prognostic correlation analysis.

The TCGA cohort was chosen for our correlation analysis between hub genes and clinical pathological features due to its relatively higher completeness of clinical information compared to the GEO cohorts. Furthermore, we utilized the “survival” package [26] to perform prognostic analysis for the hub genes.

### Quantitative real-time PCR (qRT-PCR)

Three types of PCa cells (LNCaP, PC3, and DU-145) and one type of normal prostate epithelial cell (RWPE-1) were procured from our laboratory. The cancer cell lines were cultured in 1640 medium, while RWPE-1 cells were cultured in DMEM medium. Both mediums were supplemented with 10% fetal bovine serum. When the cells reached an appropriate confluence, total cellular RNA was isolated, followed by reverse transcription into cDNA. Following mRNA quantification of the hub genes using a PCR instrument, the 2-ΔΔCT method was employed for relative expression level analysis. Supplementary Table 2 contains the primer sequences for these genes.

### Mutation and copy number alteration (CNA) analysis

We used the cbiopportal (https://www.cbioportal.org) online website for mutation and CNA analysis of hub genes. Segmentation analysis and the GISTIC algorithm were used to identify mutations and CNA in 6 hub genes [27]. The genetic alterations data for these hub genes were subsequently downloaded.

### Statistical analysis

Data analysis, statistical computations, and data visualization in this study were performed using R software (version 4.2.2) and GraphPad Prism (version 9.0). ROC curve analysis was employed to assess the diagnostic accuracy of the hub genes. Spearman's correlation coefficient, or Pearson's correlation coefficient, was utilized to determine the correlations between variables. The differential expression of the hub genes was evaluated using an unpaired t-test. A result with a p-value less than 0.05 was deemed statistically significant.

## Results

### Construction of weighted co-expression network

**Fig. 1:**
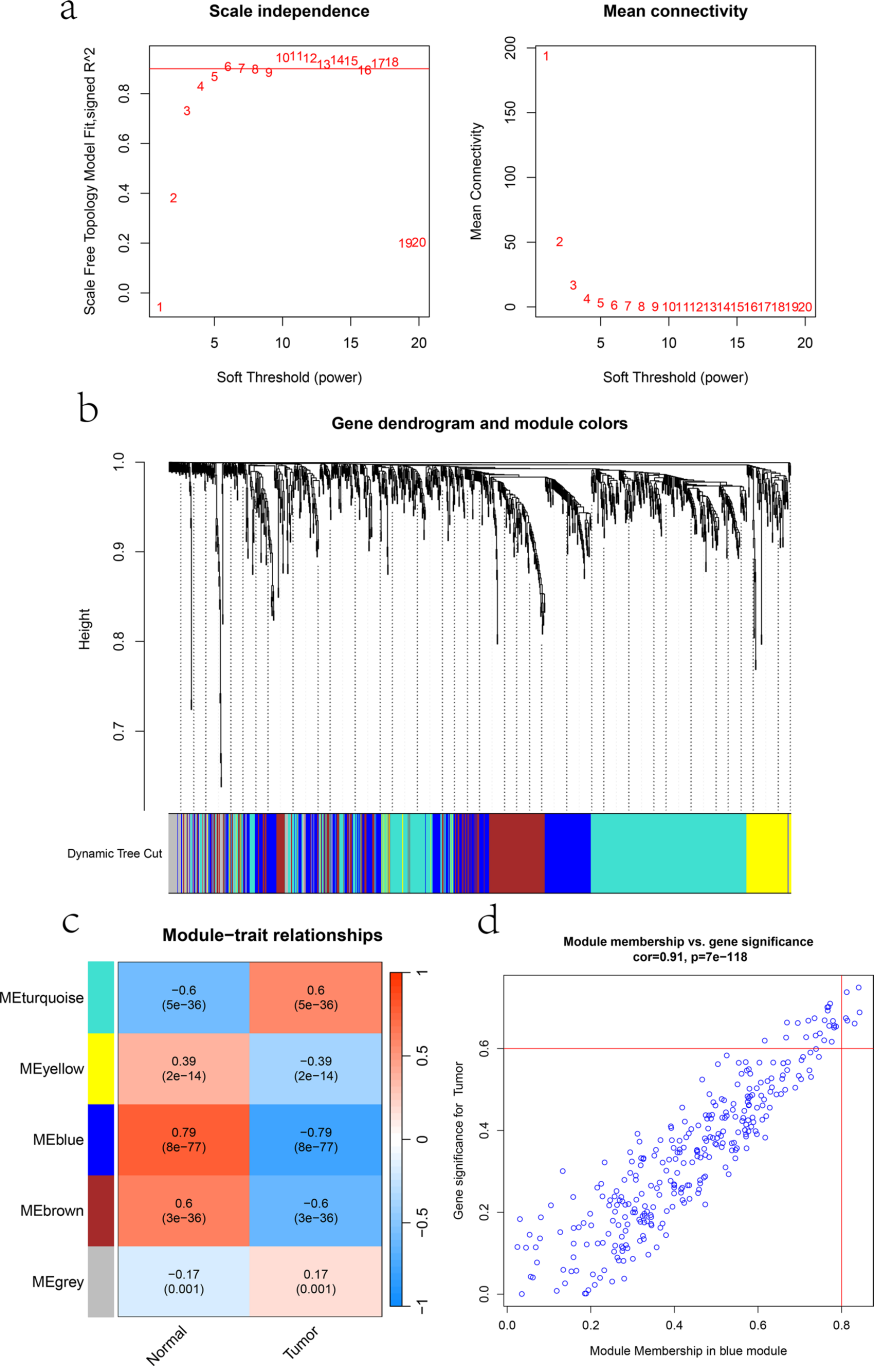
Construction of weighted co-expression network. **a** The scale-free fit index, the scale-free R^2^ is 0.85 and the soft threshold is 5. **b** The clustering dendrograms of genes, there are 5 modules. **c** The correlation of different modules with PCa traits. **d** The correlation of module membership and gene significance in the blue module

We constructed a scale-free network using a scale-free R^2^ of 0.85 and a soft threshold of 5 (Fig. 1a). Subsequently, employing hierarchical clustering analysis based on gene correlations, we successfully identified 5 distinct gene modules. (Fig. 1b). Through the analysis of the correlation between each gene module and the phenotype (PCa or control samples), we found that the blue module (cor = − 0.79, P = 8e−77) exhibited the most representative characteristics (Fig. 1c). The core genes within the blue module were identified by applying criteria with GS > 0.6 and MM > 0.8 (Fig. 1d).

### Identification of hub genes

**Fig. 2:**
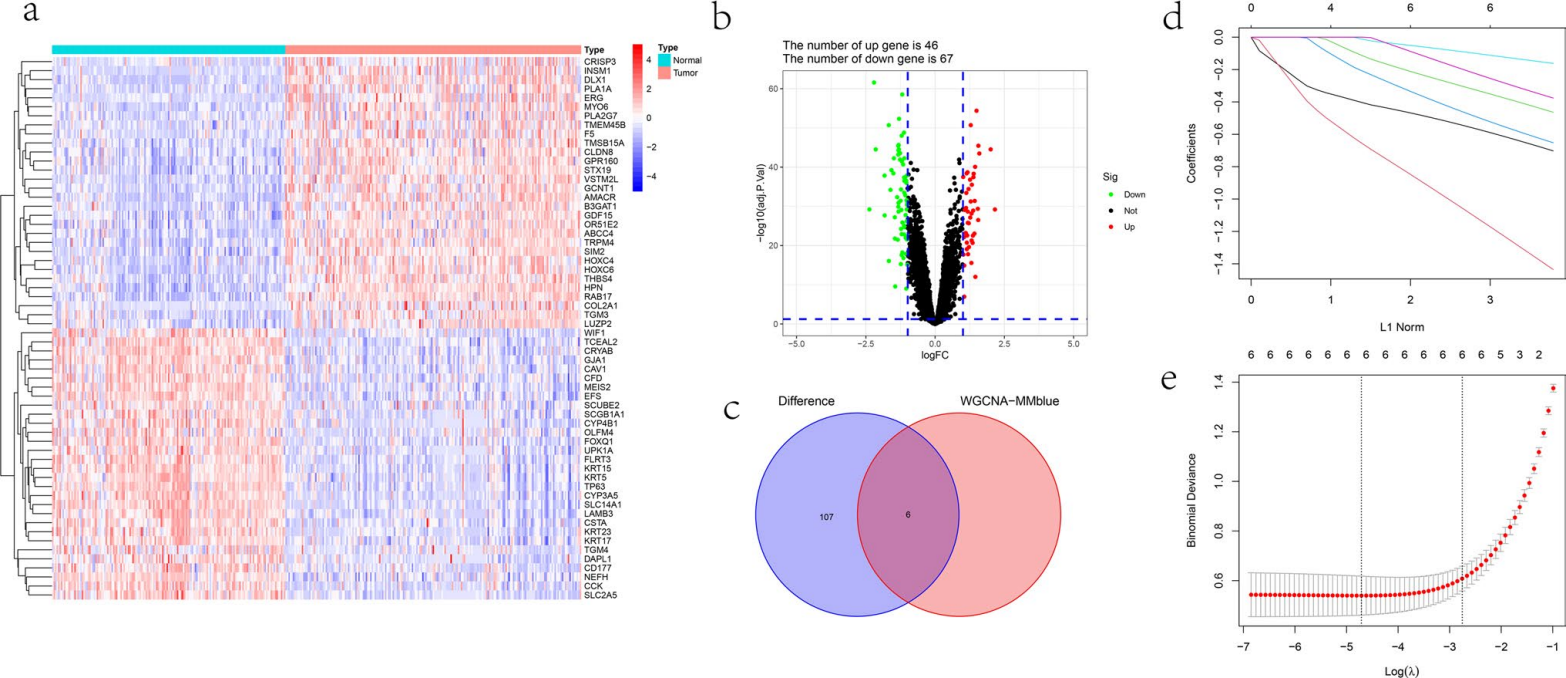
Identification of hub genes. **a** The top 30 upregulated and downregulated DEGs in GEO cohort. **b** The volcano plot of DEGs. **c** The intersection genes of core genes in the blue module and DEGs. **d** The LASSO coefficient plot of the intersection genes. **e** The selection of optimal parameter for the LASSO model

The DEGs between the tumor and normal samples were obtained through differential analysis. The heatmap displayed the top 30 upregulated and downregulated genes (Fig. 2a), and the volcano plot provided an overall overview of the DEGs, with 46 upregulated genes and 67 downregulated genes (Fig. 2b). Next, a Venn diagram was used to show the intersection genes of core genes in the blue module and DEGs (Fig. 2c). Finally, the hub genes (SLC14A1, COL4A6, MYOF, FLRT3, KRT15, and LAMB3) were identified by optimizing the fitness using the LASSO regression algorithm (Fig. 2d, e).

### Expression and verification of hub genes

**Fig. 3:**
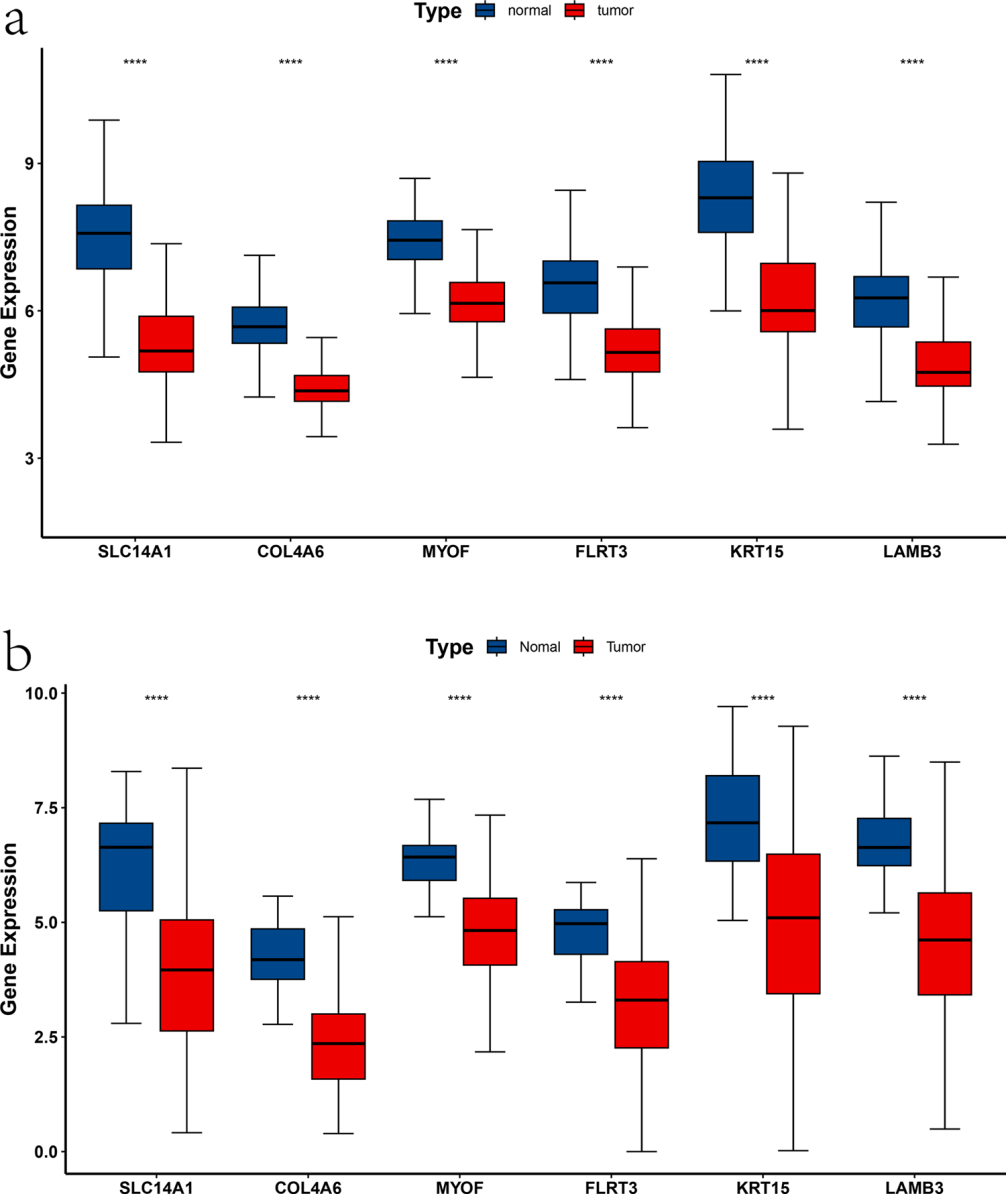
Expression of hub genes in GEO and TCGA cohorts. **a** Six hub genes in the GEO cohort are lowly expressed in tumor tissues. **b** Six hub genes in the TCGA cohort are lowly expressed in tumor tissues

**Fig. 4:**
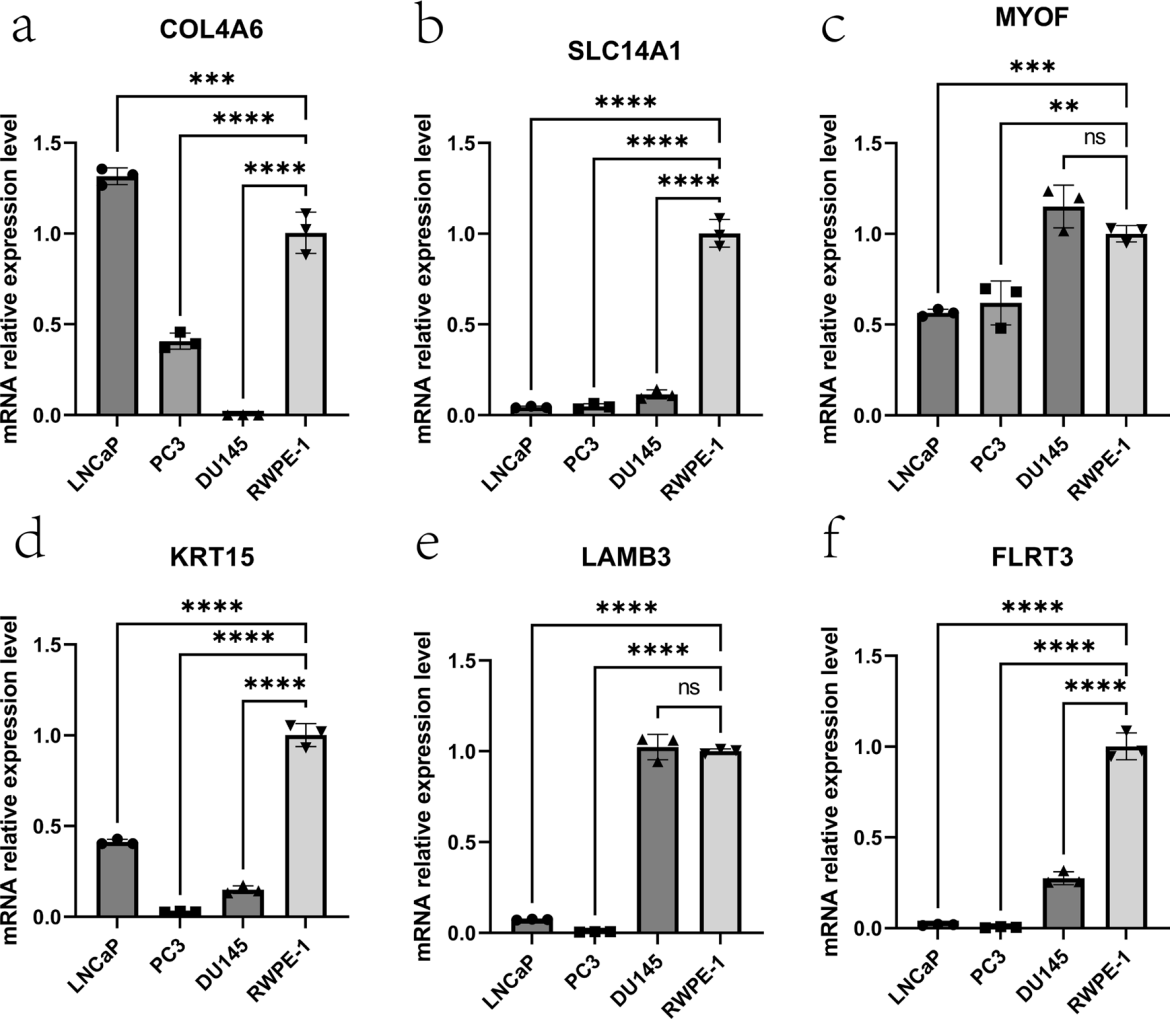
The mRNA expression levels of 6 hub genes in PCa cells (LNCaP, PC3, DU-145) and the normal prostate epithelial cell line (RWPE-1). **a**–**f** The mRNA relative expression levels of COL4A6, SLC14A1, MYOF, KRT15, LAMB3, and FLRT3 in LNCaP, PC3, DU145 and RWPE -1. The p-value of qRT-PCR for each gene can be found in Supplementary Table 4

The expression levels of the 6 hub genes were found to be consistently downregulated in PCa samples compared to normal prostate samples in the GEO dataset (Fig. 3a). Furthermore, this downregulation was validated in the TCGA dataset (Fig. 3b). Subsequently, the RNA expression levels of these genes were analyzed in three PCa cell lines (LNCaP, DU145, and PC3) and a normal prostate epithelial cell line (RWPE-1) through qRT-PCR. The results showed that these genes were also significantly downregulated in PCa cell lines compared to RWPE-1 (Fig. 4a–f). Finally, according to the immunohistochemical data existing in the HPA database, we also found that the expression of LAMB3, MYOF, FLRT3, KRT15, and SLC14A1 was lower in PCa tissues at the protein level (Supplementary Fig. 3a–e).

### Mutation and copy number alteration (CNA) analysis of 6 hub genes

We used the cBioPortal online tool to analyze the mutation status of 6 hub genes in the TCGA-PRAD cohort. The most common genetic alteration among all hub genes is Deep Deletion in PCa (Supplementary Fig. 4a). The alterations in these genes include Deep Deletion, Missense Mutation, Missense Mutation, and Amplification, in which each hub gene undergoes Deep Deletion (Supplementary Fig. 4b). MYOF (5%) has the highest rate of genetic alteration among these hub genes (Supplementary Fig. 4b).

### Evaluation of hub genes for disease diagnosis

**Fig. 5:**
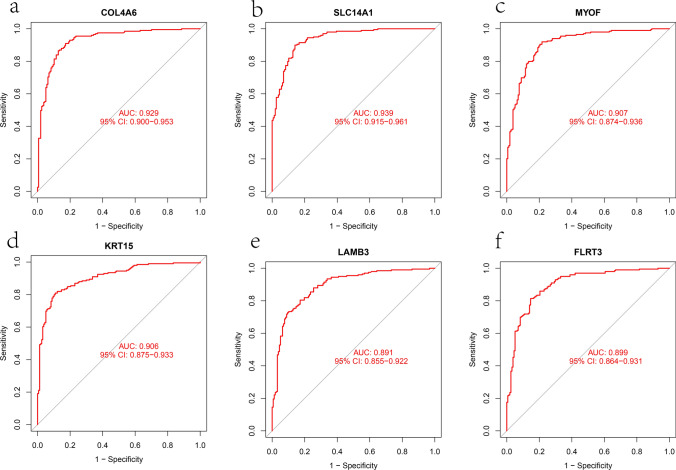
The diagnostic efficacy of six hub genes was confirmed by ROC curve analysis based on GEO cohort. **a**–**f** The ROC curve of COL4A6, SLC14A1, MYOF, KRT15, LAMB3, and FLRT3

**Fig. 6:**
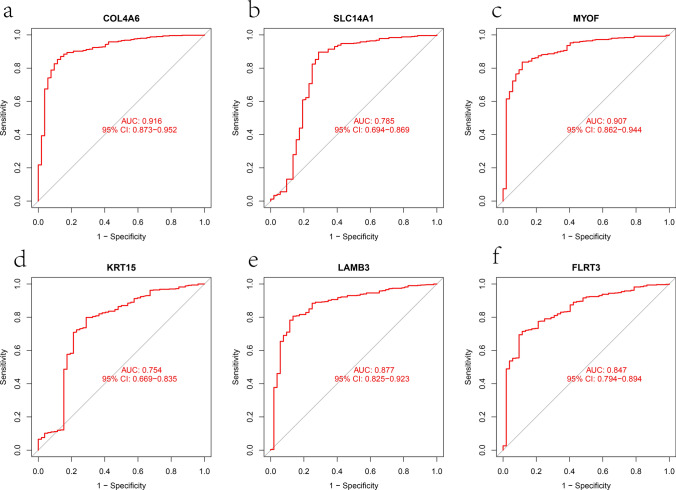
The diagnostic efficacy of six hub genes was confirmed by ROC curve analysis based on TCGA cohort. **a**–**f** The ROC curve of COL4A6, SLC14A1, MYOF, KRT15, LAMB3, and FLRT3

In order to evaluate the diagnostic ability of the hub genes, we performed ROC curve analysis on each gene in the GEO cohort. The AUC for COL4A6 was 0.929 (Fig. 5a), SLC14A1 was 0.939 (Fig. 5b), MYOF was 0.907 (Fig. 5c), KRT15 was 0.906 (Fig. 5d), LAMB3 was 0.891 (Fig. 5e), and FLRT3 was 0.899 (Fig. 5f), respectively. Furthermore, in the validation cohort using the TCGA dataset, the AUC for COL4A6 was 0.916 (Fig. 6a), SLC14A1 was 0.785 (Fig. 6b), MYOF was 0.907 (Fig. 6c), KRT15 was 0.754 (Fig. 6d), LAMB3 was 0.877 (Fig. 6e), and FLRT3 was 0.847 (Fig. 6f). These results suggest that these hub genes are promising diagnostic biomarkers for PCa.

**Fig. 7:**
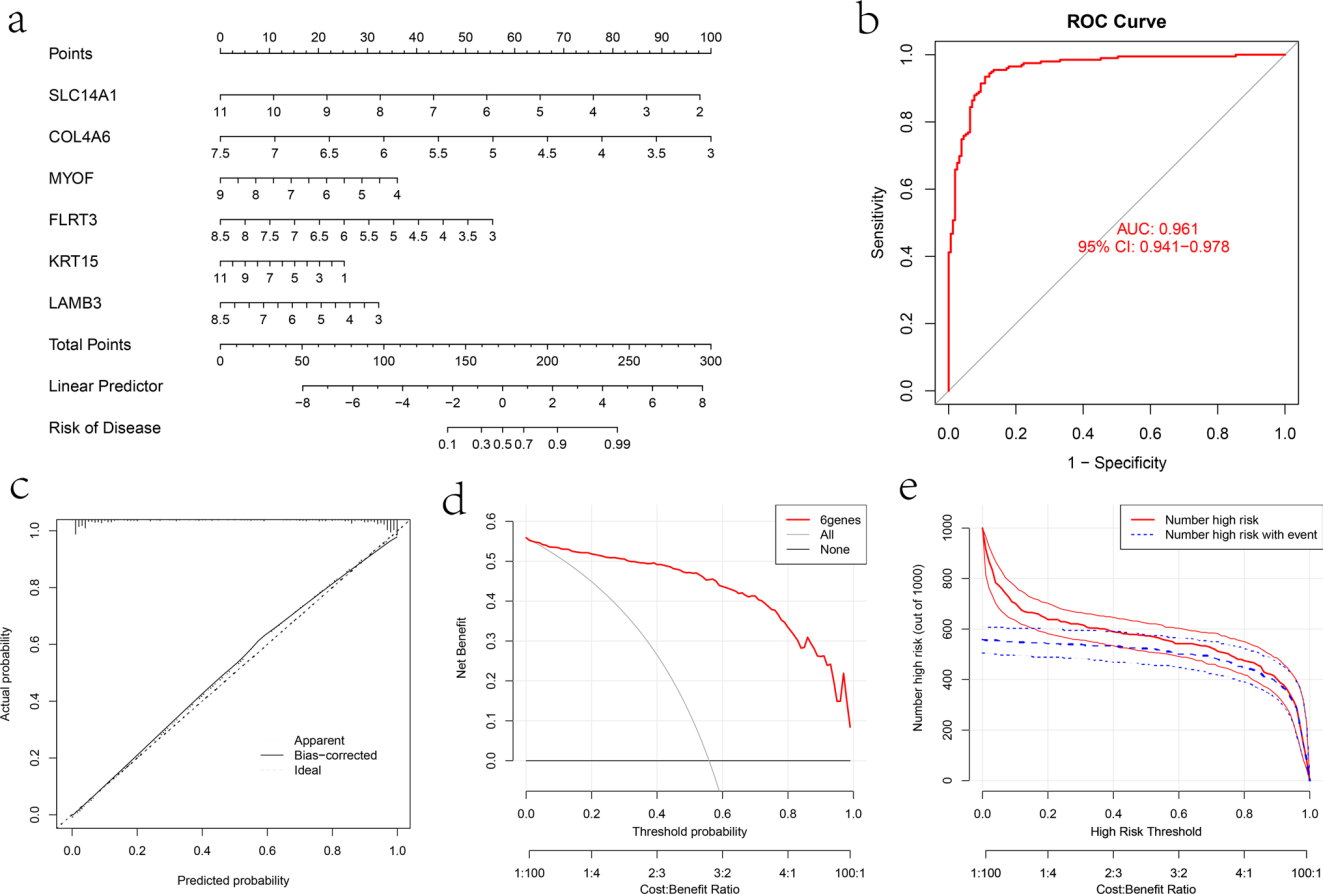
Construction and evaluation of nomogram for diagnostic prediction. **a** The nomogram for predicting disease risk based on 6 key genes. **b** The ROC curve of this nomogram model. **c** The calibration curve of the nomogram model. **d** The DCA curve of the nomogram model. **e** The clinical impact curve of the nomogram model

To assess the overall predictive ability of the hub genes, we constructed a nomogram to predict the risk of disease occurrence using these 6 hub genes (Fig. 7a). In this nomogram, the corresponding score of each gene can be obtained according to the expression of hub genes, and the incidence rate of PCa can be judged based on the sum of the obtained scores. In order to verify the accuracy and clinical benefit of the nomogram, the ROC curve shows that the AUC of the nomogram is 0.961 (Fig. 7b), the calibration curve shows that the predicted curve is basically consistent with the actual curve (Fig. 7c), the DCA curve shows that the nomogram model curve is far away from the other two curves (Fig. 7d), and the clinical impact curve shows that at a high risk threshold of 0.2 to 1, the two curves are close (Fig. 7e). These results showed that the PCa prediction nomogram constructed based on 6 hub genes had high prediction efficiency.

### Gene correlation analysis and GSEA function analysis

**Fig. 8:**
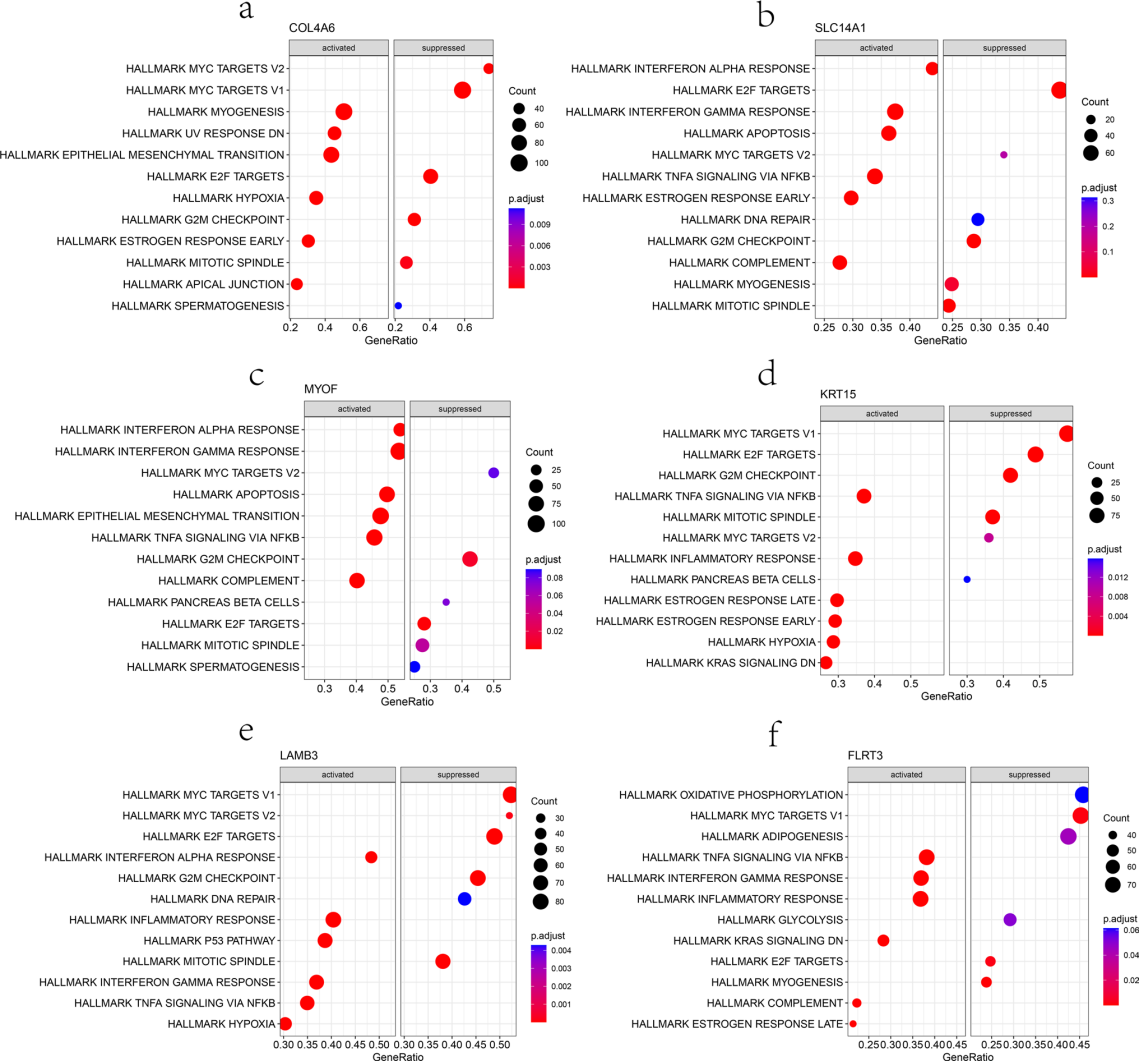
GSEA function analysis of these six hub genes based on hallmark gene sets. **a**–**f** The GSEA analysis of COL4A6, SLC14A1, MYOF, KRT15, LAMB3, and FLRT3

Firstly, through correlation analysis of these 6 key genes, it was found that they are positively correlated (Supplementary Fig. 1a). In addition, Gene Set Enrichment Analysis (GSEA) was conducted for each gene, and the results indicate that the pathways inhibited by COL4A6 include MYC targets V1/V2, E2F targets, G2M checkpoint, and mitotic spindle (Fig. 8a). The pathways inhibited by SLC14A1 include E2F targets, G2M checkpoint, mitotic spindle, DNA repair, and MYC targets V1 (Fig. 8b). The pathways inhibited by MYOF include E2F targets, G2M checkpoint, mitotic spindle, and MYC targets V2 (Fig. 8c). The pathways inhibited by KRT15 include MYC targets V1/V2, E2F targets, G2M checkpoint, and mitotic spindle (Fig. 8d). The pathways inhibited by LAMB3 include MYC targets V1/V2, E2F targets, G2M checkpoint, mitotic spindle, and DNA repair (Fig. 8e). The pathways inhibited by FLRT3 include MYC targets V1, E2F targets, and others (Fig. 8f). Previous studies have shown that these gene sets, including MYC targets V1/V2, E2F targets, G2M checkpoint, mitotic spindle, and DNA repair, are related to cancer cell proliferation, apoptosis, tumor growth, and tumor metastasis. Therefore, it can be inferred that the downregulation of these 6 hub genes in PCa may weaken the inhibition of these functions and promote tumor occurrence and development.

### Clinical correlation analysis

**Fig. 9:**
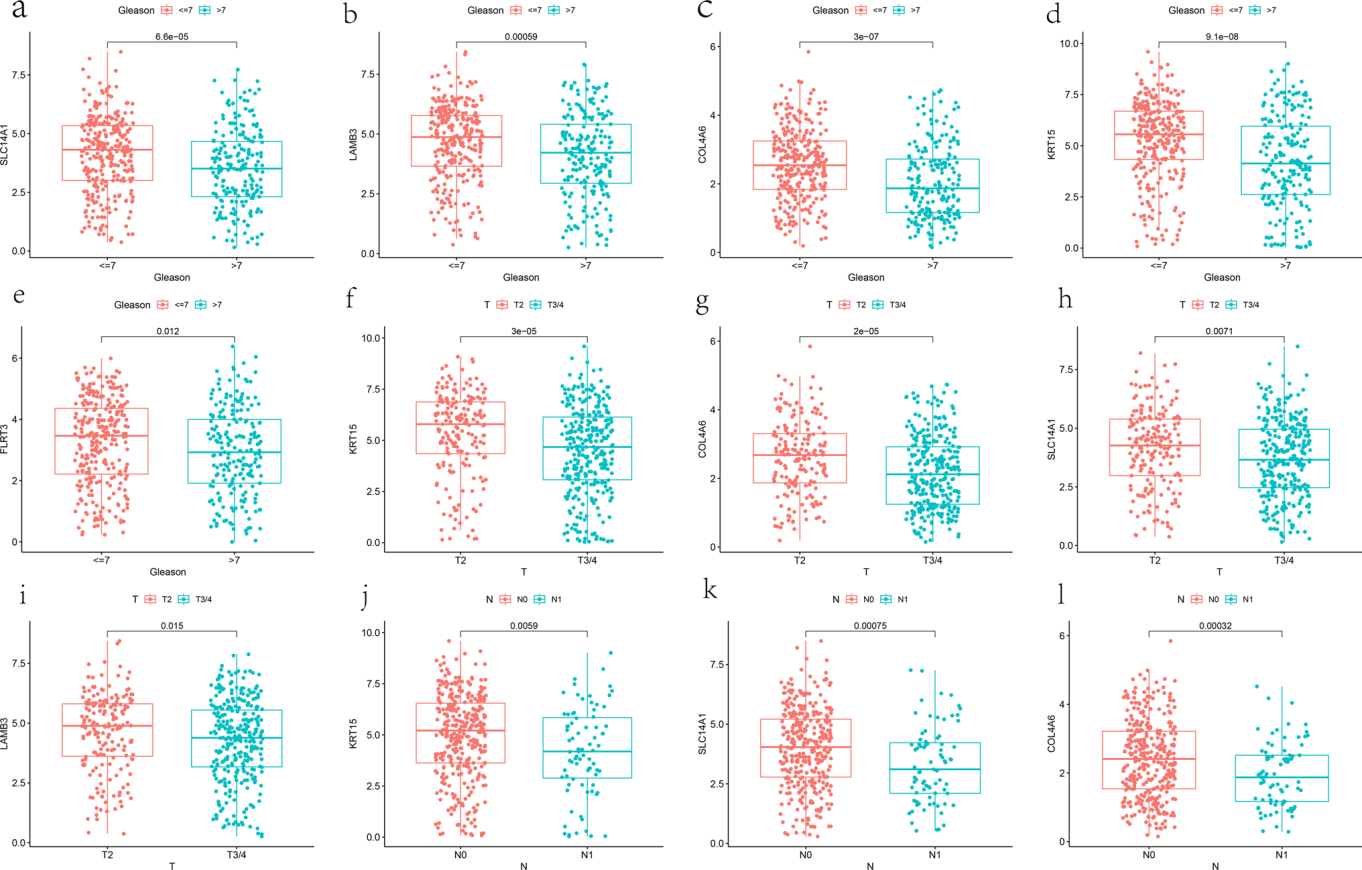
The correlation between hub genes and clinical features of PCa. **a**–**e** The expression levels of SLC14A1, LAMB3, COL4A6, KRT15, and FLRT3 were lower in patients with Gleason score > 7. **f**–**i** The expression of SLC14A1, COL4A6, KRT15, and LAMB3 was lower in patients with T3 and T4 stages. **j**–**l** The expression of SLC14A1, COL4A6, and KRT15 was lower in patients with N1 stage

**Fig. 10:**
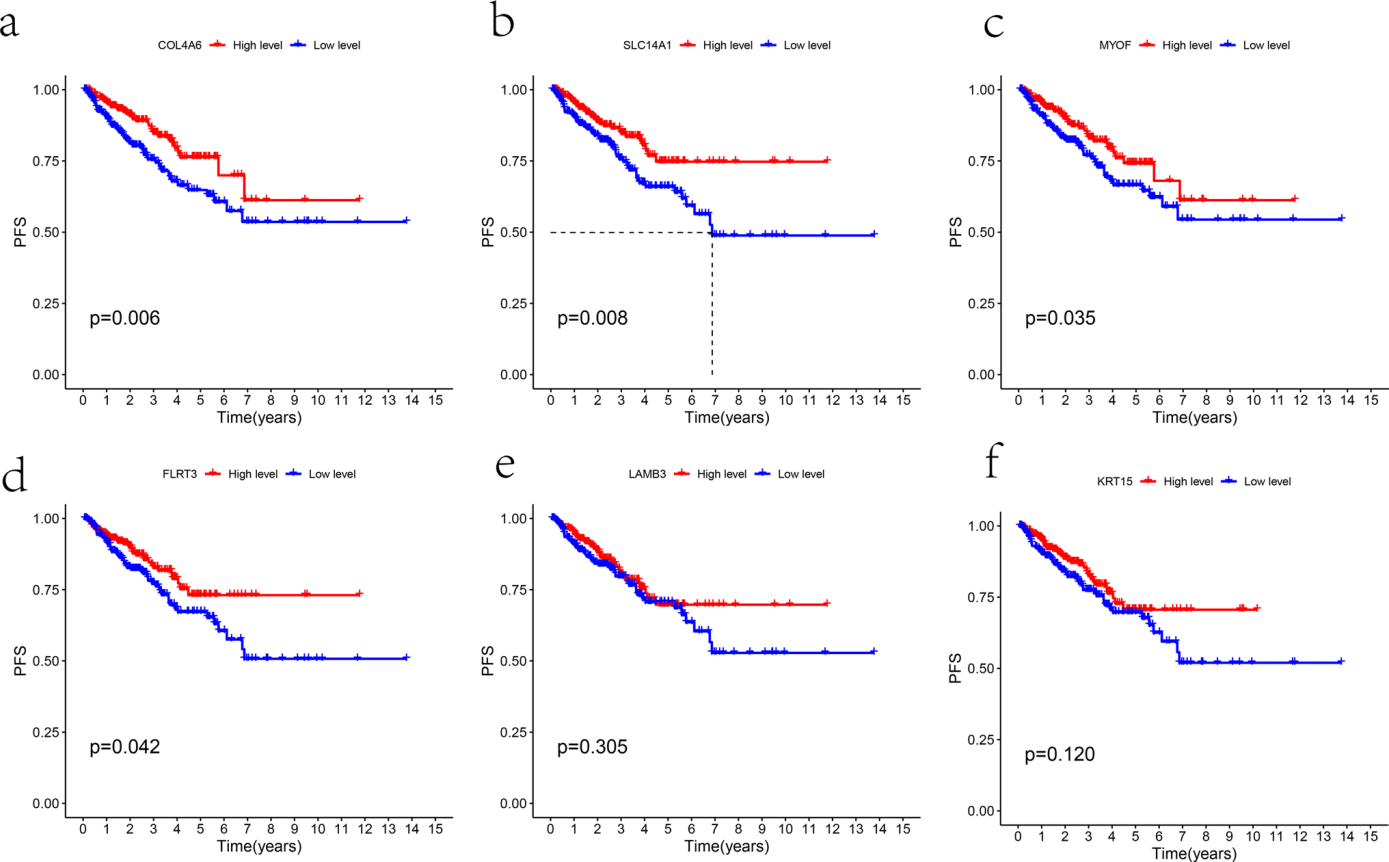
The survival analysis of 6 hub genes. **a**–**f** Patients with lower expression levels of COL4A6, SLC14A1, MYOF, KRT15, LAMB3, and FLRT3 exhibited a worse progression-free survival (PFS)

Some studies have shown that patients with Gleason score ≤ 7 are considered low-risk for PCa [28]. In addition, according to the recommendations of the American Joint Committee on Cancer (AJCC) pathological TNM classification of PCa, T2 stage tumors are limited to the prostate, while T3/4 stage tumors begin to invade tissues outside the prostate. Compared with stage N0 tumors, stage NI tumors have metastasized to lymph nodes [29]. Therefore, patients with T3/4 and N1 stages mean that their tumors have progressed or metastasized to a certain extent, and they may have a poor prognosis. In this study, due to the availability of comprehensive clinical information in the TCGA database for PCa patients, we analyzed the correlation between these 6 hub genes and corresponding clinical pathological features such as age, T/N stage, and Gleason score. The information included in the study can be found in Supplementary Table 3. The results showed that the expression of SLC14A1, COL4A6, FLRT3, KRT15, and LAMB3 was lower in patients with Gleason score > 7 (Fig. 9a–e). Similarly, the expression of SLC14A1, COL4A6, KRT15, and LAMB3 was also lower in patients with T3 and T4 stages (Fig. 9f–i). In patients with N1 stage, the expression of SLC14A1, COL4A6, and KRT15 was lower as well (Fig. 9j–l). These results showed significant differences (p < 0.05). Additionally, Supplementary Fig. 2, which includes data with p > 0.05, also suggests that the expression of these 6 hub genes was relatively lower in patients with Gleason score > 7, T3/4 stages, N1 stage, and age ≥ 60. Finally, the survival analysis revealed that patients with lower expression levels of the six hub genes exhibited a worse progression-free survival (PFS) outcome (Fig. 10a–f).

### Immune infiltration analysis and drug sensitivity prediction

**Fig. 11:**
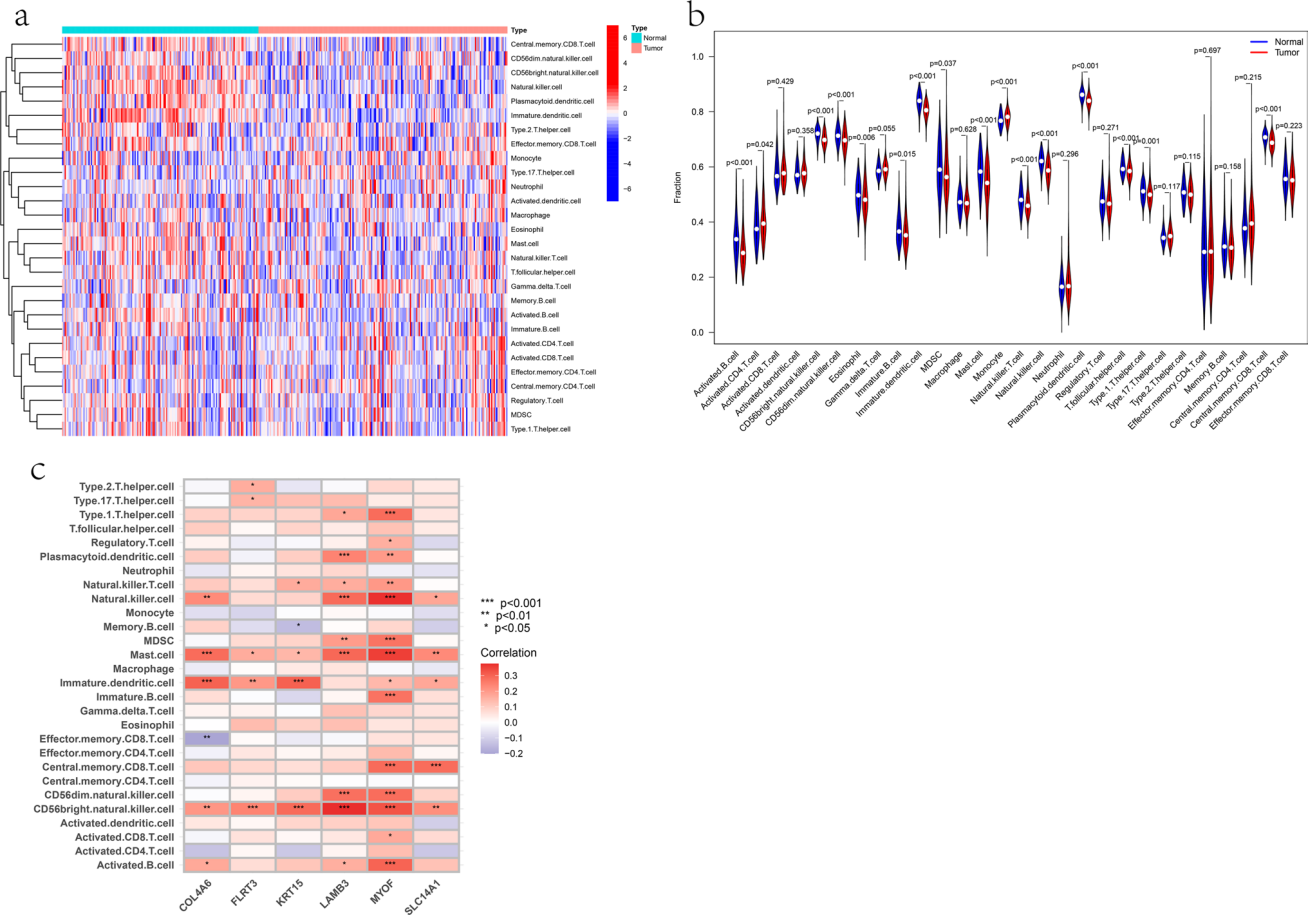
Immune infiltration analysis. **a** The heatmap of immune cell infiltration between PCa tumors and normal samples. **b** The violin plot of immune cell infiltration between PCa tumors and normal samples. **c** The correlation between 6 hub genes and immune cell infiltration

We conducted single-sample gene set enrichment analysis (ssGSEA) to analyze the infiltration of immune cells in PCa and normal prostate samples from the GEO dataset. The results showed that compared to normal samples, PCa samples had lower levels of Activated B cells, CD56(bright) natural killer cells, CD56(dim) natural killer cells, Eosinophils, Immature B cells, Immature dendritic cells, Myeloid-derived suppressor cells (MDSCs), Mast cells, Natural killer T cells, Natural killer cells, Plasmacytoid dendritic cells, T follicular helper cells, Type 1T helper cells, and Central memory CD8 T cells (Fig. 11a, b), while levels of Activated CD4 T cells and Monocytes were higher (Fig. 11a, b). The correlation analysis of the 6 key genes with immune cell infiltration showed that they were positively correlated with the infiltration of most immune cells (Fig. 11c), which is consistent with their low expression in tumor tissues.

**Fig. 12:**
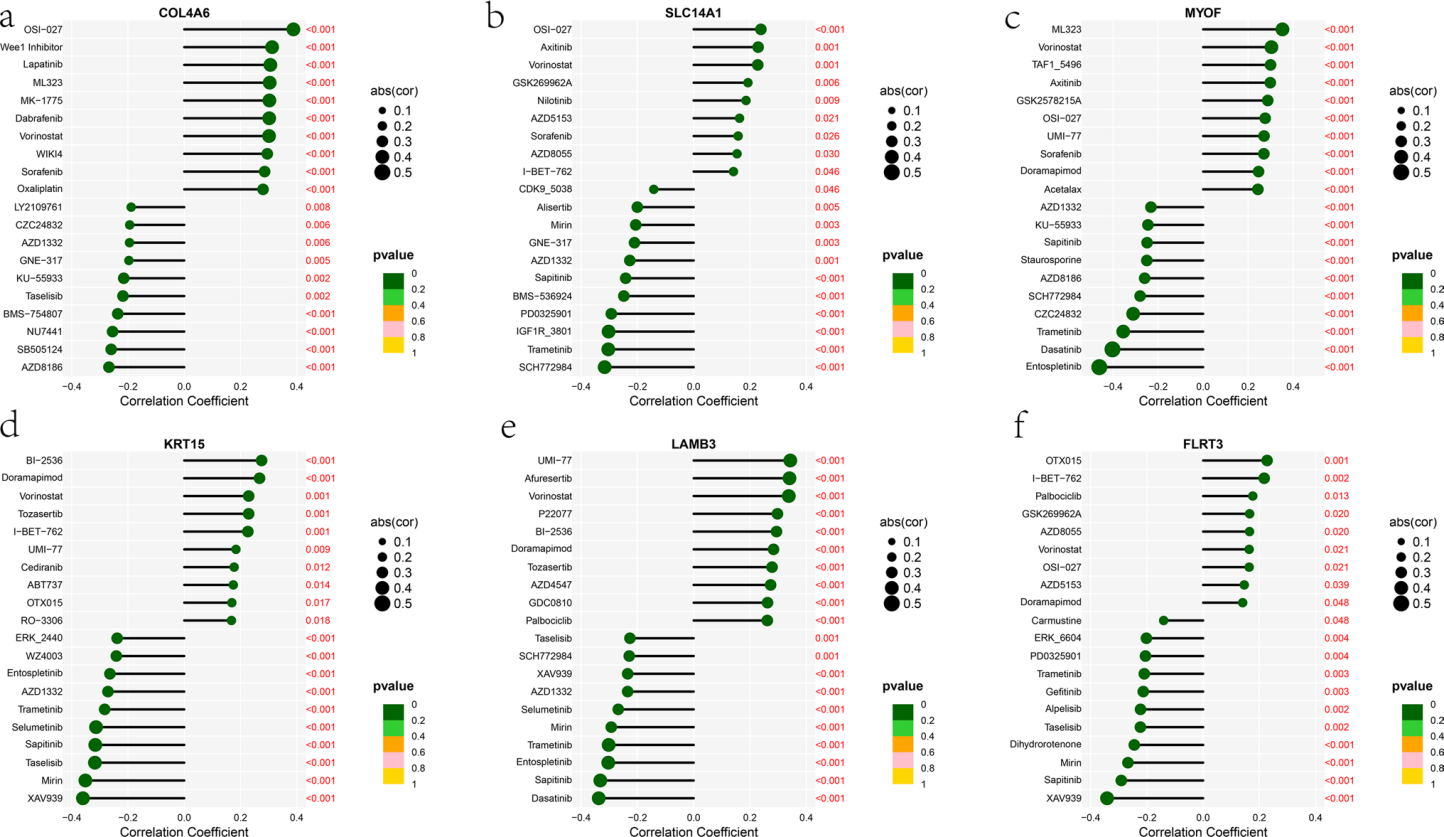
Drug sensitivity analysis of the six hub genes. **a**–**f** The correlation between six hub genes and drug sensitivity

In order to further investigate the potential application of hub genes in personalized therapy for PCa, we evaluated their correlation with the IC50 values of drugs in the GDSC database. We presented the drugs that were most correlated with these 6 hub genes. For example, COL4A6 showed a positive correlation with the IC50 of OSI-027 and a negative correlation with the IC50 of AZD8186 (Fig. 12a). SLC14A1 showed a positive correlation with the IC50 of OSI-027 and a negative correlation with the IC50 of SCH772984 (Fig. 12b). MYOF showed a positive correlation with the IC50 of ML323 and a negative correlation with the IC50 of Entospletinib (Fig. 12c). KRT15 showed a positive correlation with the IC50 of BI-2536 and a negative correlation with the IC50 of XAV939 (Fig. 12d). LAMB3 showed a positive correlation with the IC50 of UMI-77 and a negative correlation with the IC50 of Dasatinib (Fig. 12e). FLRT3 showed a positive correlation with the IC50 of OTX015 and a negative correlation with the IC50 of XAV939 (Fig. 12f). These results suggest that the expression of these key genes might help patients benefit from relevant drug treatments.

## Discussion

Prostate cancer (PCa) is a prevalent cancer among men and a leading cause of cancer-related mortality. When PCa becomes castration-resistant, it is typically incurable [30]. Therefore, an early and accurate diagnosis of PCa is crucial for effective treatment. However, due to the significant heterogeneity of prostate tumors in terms of clinicopathological, molecular, and morphological characteristics, the diagnosis and treatment of PCa still pose challenges [31]. Therefore, the identification of new diagnostic biomarkers for PCa remains a critical area of research for improving the treatment and prognosis of this disease.

The weighted gene co-expression network analysis (WGCNA) method has been widely used in various diseases to identify common biomarkers and pathways associated with different disease phenotypes [32]. In this study, we utilized the WGCNA method to construct a co-expression network to investigate the expression profiles of hub genes related to PCa. We obtained data sets (GSE88808, GSE69223, GSE46602, GSE32571, and GSE32448) from the GEO database and performed WGCNA analysis to identify modules of co-expressed genes. Among these modules, we found a significant correlation between the blue module and prostate tumor samples. Further differential gene expression analysis and LASSO regression analysis led to the identification of 6 hub genes that are potentially associated with PCa. According to reports, Collagen Type IV Alpha 6 Chain (COL4A6) is a prominent constituent of the basement membrane, and it has been observed that hypermethylation of the COL4A6 gene promoter is associated with decreased expression in PCa. Furthermore, the downregulation of COL4A6 may potentially facilitate the progression and invasion of PCa by activating the p-FAK/MMP-9 signaling pathway [33]. Solute Carrier Family 14 Member 1 (SLC14A1) encodes urea transporter protein B (UT-B), which can affect urea levels and lead to metabolic disorders, and is believed to be associated with the occurrence and development of urothelial carcinoma of the urinary tract [34]. SLC14A1 is downregulated in PCa and plays an important role in the biochemical recurrence of PCa [35]. Laminin Subunit Beta 3 (LAMB3) encodes laminin subunit beta-3, a protein involved in important biological behaviors such as cell differentiation, adhesion, and survival, and is associated with the metastatic ability of various cancers such as colorectal cancer, pancreatic cancer, thyroid cancer, and lung cancer [36, 37]. LAMB3 has been shown to be downregulated in PCa cell lines [38]. FLRT3 is a transmembrane protein that belongs to the axon guidance molecule family [39]. FLRT3 is involved in cell adhesion and adipokine signaling pathways and has inhibitory effects on tumorigenesis and metastasis [40]. Keratin-15 (KRT15) is crucial for maintaining cytoplasmic stability and may serve as a new marker for urothelial precursor cells [41], with low expression confirmed in PCa [42]. MYOF (Myoferlin), as a key vesicle transport protein, has become an attractive target for cancer therapy. MYOF is related to multiple biological processes such as cell membrane shape maintenance, cell migration, and endoplasmic reticulum stress [43]. MYOF may play an important role in the migration, invasion, and metastasis of tumor cells [44]. These hub genes may serve as promising biomarkers for PCa and provide valuable insights into the molecular mechanisms underlying PCa development and progression.

In order to validate the accuracy of these hub genes as biomarkers for PCa diagnosis, we found that they were downregulated in tumor tissues in both the GEO and TCGA validation cohorts, which was further confirmed by qRT-PCR in vitro cell experiments. Meanwhile, we analyzed possible genetic alterations in these hub genes through cBioPortal and found that Deep Deletion has occurred in these genes, and Deep Deletion is the most common genetic alteration in these hub genes. And previous studies indicated that the occurrence of Deep Deletion in the genome may be an important cause of gene downregulation [45]. Therefore, we speculate that the downregulation of these hub genes is related to the occurrence of Deep Deletion of genes. The results of ROC curve analysis indicated that they had high value as biomarkers for PCa diagnosis, and the nomogram plot demonstrated their excellent efficacy in predicting PCa risk. Additionally, we found that the expression of these six genes was lower in PCa patients with higher T/N pathological stage and Gleason score, and patients with low expression of these genes may have poorer progression-free survival (PFS). Furthermore, these six genes were positively correlated with each other, and the GSEA revealed that they played inhibitory roles in pathways or functions related to PCa growth and metastasis, such as MYC targets V1/V2, E2F targets, G2M checkpoint, mitotic spindle, and DNA repair. Due to their downregulation in PCa, the inhibition of these pathways or functions may be weakened, thus promoting tumor initiation and progression. Based on previous reports and our research, these hub genes can be used as prognostic biomarkers for early diagnosis and disease progression of PCa and may be potential therapeutic targets for PCa.

Subsequent analysis of immune cell infiltration in PCa samples revealed a decreased infiltration level of Activated B cell, CD56(bright) natural killer cell, CD56(dim) natural killer cell, Eosinophil, Immature B cell, Immature dendritic cell, MDSC, Mast cell, Natural killer T cell, Natural killer cell, Plasmacytoid dendritic cell, T follicular helper cell, Type 1T helper cell, and Central memory CD8 T cell. Conversely, a markedly elevated infiltration level of Activated CD4 T cell and Monocyte was observed. Based on previous investigations, our findings suggested that despite the presence of numerous tumor-associated antigens in PCa that could potentially serve as targets for immunotherapy, the limited infiltration of immune cells into the tumor microenvironment results in a restricted response to immunotherapeutic interventions [46, 47]. Furthermore, it was observed that these six hub genes exhibited a positive correlation with the infiltration of various immune cell types, which was consistent with their low expression levels in PCa samples. The identification of this association between the hub genes and immune cell infiltration may offer novel insights and potential strategies for improving immunotherapeutic approaches for PCa. Finally, the examination of the correlation between these genes and drug sensitivity in PCa may yield valuable insights into the precise treatment of this malignancy and potentially facilitate the development of combination treatment strategies involving chemotherapy drugs, immunotherapy, and hormone therapy for PCa.

The objective of this study was to identify novel diagnostic biomarkers for PCa and investigate their associations with clinicopathological features, prognosis, immune infiltration, and drug sensitivity. Our findings were validated through a comprehensive analysis of multiple databases, which demonstrated promising results for the diagnosis of PCa. However, several limitations need to be acknowledged. Firstly, potential selection bias may have existed during the dataset selection process, despite efforts to minimize it through the use of multiple machine learning methods. Secondly, to confirm the accuracy of our identified biomarkers, further prospective multicenter studies with larger sample sizes are warranted. Additionally, functional experiments involving in vitro and in vivo investigations are necessary to elucidate the underlying molecular mechanisms by which the identified genes may impact PCa.

## Conclusion

In summary, our study has identified six hub genes, namely SLC14A1, COL4A6, MYOF, FLRT3, KRT15, and LAMB3, which can be utilized to establish a diagnostic model for PCa. Additionally, our investigation has revealed novel insights into their association with the immune microenvironment and drug sensitivity in PCa. The discovery of these genes may offer potential molecular targets for clinical diagnosis and treatment of PCa.

## Supplementary Information

Supplementary file 1 Table 1: Information on microarray datasets obtained from GEO and TCGA databases. Table 2: The primer sequence for all the genes. Table 3: The clinical information of patients in TCGA database. Table 4: The p-value of qRT-PCR for each gene. Figure 1: Correlation of the six hub gene. Figure 2: The correlation between hub genes and clinical features of PCa. Figure 3: Immunohistochemistry data of hub genes from the HPA database. Figure 4: Mutation and copy number alteration (CNA) analysis of 6 hub genes (DOCX 59716 KB).

## Data Availability

The original contributions presented in the study are included in the article/Supplementary Material, further inquiries can be directed to the corresponding authors.
