# Supplementary material for: Identification and validation of diagnostic and prognostic biomarkers in prostate cancer based on WGCNA
Source: Discov Oncol. 2024 Sep 21;15:131. doi: 10.1007/s12672-024-00983-5 (PMC11415334; doi:10.1007/s12672-024-00983-5)
Supplement: Supplementary file 1 — Table 1: Information on microarray datasets obtained from GEO and TCGA databases. Table 2: The primer sequence for all the genes. Table 3: The clinical information of patients in TCGA database. Table 4: The p-value of qRT-PCR for each gene. Figure 1: Correlation of the six hub gene. Figure 2: The correlation between hub genes and clinical features of PCa. Figure 3: Immunohistochemistry data of hub genes from the HPA database. Figure 4: Mutation and copy number alteration (CNA) analysis of 6 hub genes (DOCX 59716 KB). [file 12672_2024_983_MOESM1_ESM.docx]

Supplementary Materials

# Supplementary Tables

Supplementary Table 1: Information on microarray datasets obtained from GEO and TCGA databases.

| Datasets | Type | Sample size | | Platform | Release time | Acquisition time |
| --- | --- | --- | --- | --- | --- | --- |
|  |  | Normal | Tumor |  |  |  |
| GSE88808 | mRNA | 49 | 49 | GPL22571 | Nov 30, 2016 | Jun 20,2022 |
| GSE69223 | mRNA | 15 | 15 | GPL570 | Apr 22, 2018 | Jun 20,2022 |
| GSE46602 | mRNA | 14 | 36 | GPL570 | Jul 01, 2015 | Jun 20,2022 |
| GSE32571 | mRNA | 39 | 59 | GPL6947 | Oct 19, 2012 | Jun 20,2022 |
| GSE32448 | mRNA | 40 | 40 | GPL570 | Sep 29, 2011 | Jun 20,2022 |
| TGCA-PRAD | mRNA | 52 | 501 | - | Continually updated | Jun 20,2022 |

Supplementary Table 2: The primer sequence for all the genes.

| Primer | Sequence (5'to3') |
| --- | --- |
| AURKB-F | GGGAGAGCTGAAGATTGCTG |
| AURKB-R | GGCGATAGGTCTCGTTGTGT |
| SLC14A1-F | TGGCAAACTTTATGGCTGAGG |
| SLC14A1-R | GAACAATAGCGTGGCCAAACA |
| COL4A6-F | GGATTGCCAGCA TTATCAGGT |
| COL4A6-R | GTCTCAAATTCTGGACTAGGTGG |
| MYOF-F | CAAGCTGATCTCCCTGCTAAA |
| MYOF-R | ACCTGTCTTCATCACCTTCATC |
| KRT15-F | AGACCTGAGACGCACGATG |
| KRT15-R | CGGTAAGTAGCGATCTCCTGC |
| LAMB3-F | AGGAGCTTTCAGGCGATCTG |
| LAMB3-R | CCCCAACAGGTGGATAGCAG |
| FLRT3-F | TTCTCGTCTTCCTGGGTTCTG |
| FLRT3-R | GTCCCGATGAGGAAGATGCTC |
| ACTB-F | CCAACCGCGAGAAGATGACC |
| ACTB-R | AGCACAGCCTGGATAGCAAC |

F: forward R: reverse

Supplementary Table 3: the clinical information of patients in TCGA database.

| Variables | Type | Number |
| --- | --- | --- |
| Age | <60 | 202(40.64%) |
|  | >=60 | 295(59.36%) |
| T stage | T2 | 187(37.63%) |
|  | T3/4 | 303(60.97%) |
|  | unknow | 7(1.40%) |
| N stage | N0 | 345(49.42%) |
|  | N1 | 79(15.89%) |
|  | unknow | 73(14.69%) |
| Gleason score | <=7 | 292(58.75%) |
|  | >7 | 205(41.25%) |

Supplementary Table 4: the p-value of qRT-PCR for each gene.

| Hub-genes | Comparison among groups | P-value |
| --- | --- | --- |
| LAMB3 | RWPE-1 vs. LNCaP | <0.0001 |
|  | RWPE-1 vs. PC3 | <0.0001 |
|  | RWPE-1 vs. DU145 | 0.7676 |
| MYOF | RWPE-1 vs. LNCaP | 0.0008 |
|  | RWPE-1 vs. PC3 | 0.0018 |
|  | RWPE-1 vs. DU145 | 0.1608 |
| COL4A6 | RWPE-1 vs. LNCaP | 0.0010 |
|  | RWPE-1 vs. PC3 | <0.0001 |
|  | RWPE-1 vs. DU145 | <0.0001 |
| KRT15 | RWPE-1 vs. LNCaP | <0.0001 |
|  | RWPE-1 vs. PC3 | <0.0001 |
|  | RWPE-1 vs. DU145 | <0.0001 |
| FLRT3 | RWPE-1 vs. LNCaP | <0.0001 |
|  | RWPE-1 vs. PC3 | <0.0001 |
|  | RWPE-1 vs. DU145 | <0.0001 |
| SLC14A1 | RWPE-1 vs. LNCaP | <0.0001 |
|  | RWPE-1 vs. PC3 | <0.0001 |
|  | RWPE-1 vs. DU145 | <0.0001 |

# Supplementary Figures

Supplementary Figure 1
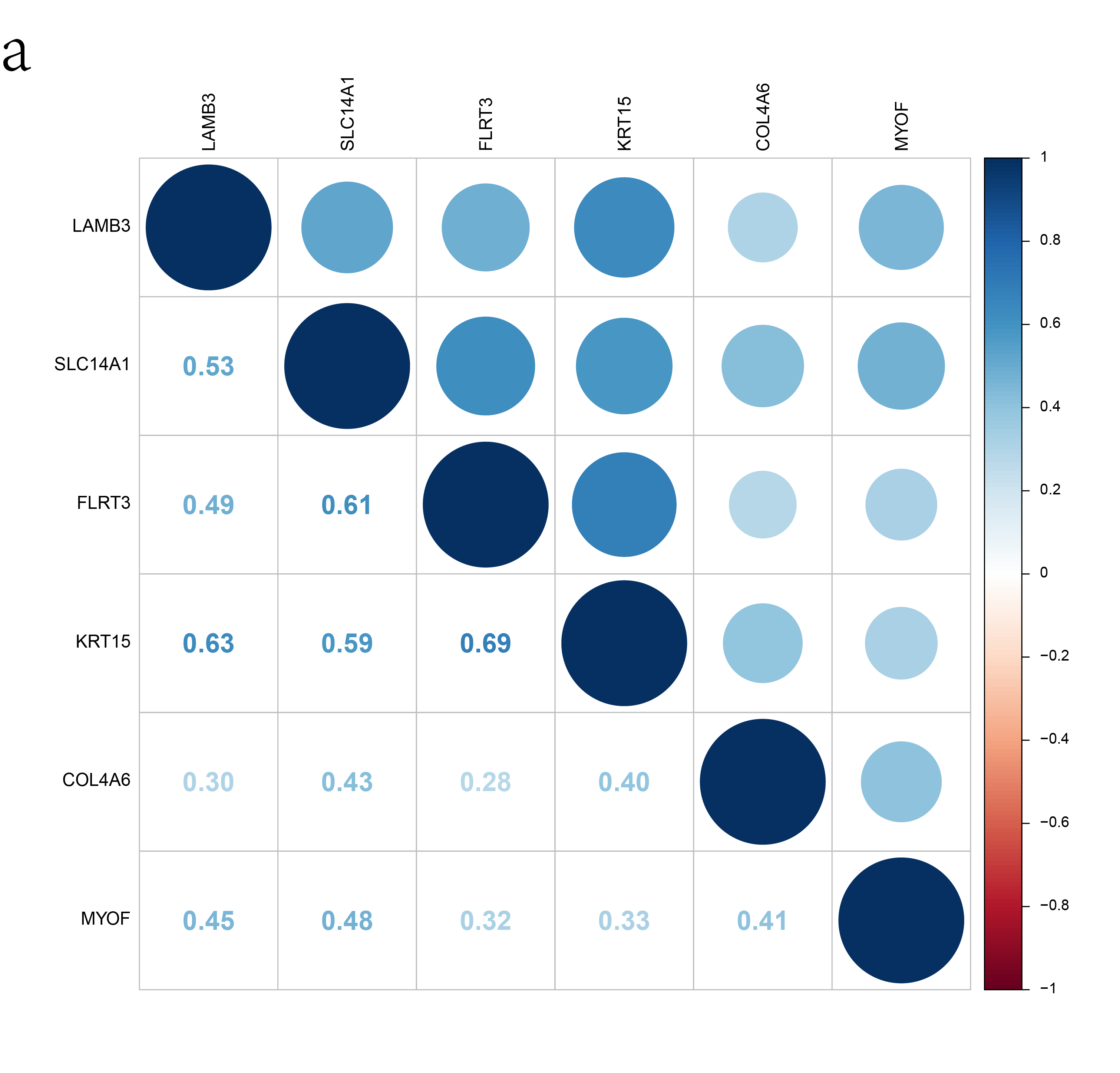


Supplementary Figure 1: Correlation of the six hub genes (COL4A6, SLC14A1, MYOF, KRT15, LAMB3, and FLRT3).

Supplementary Figure 2


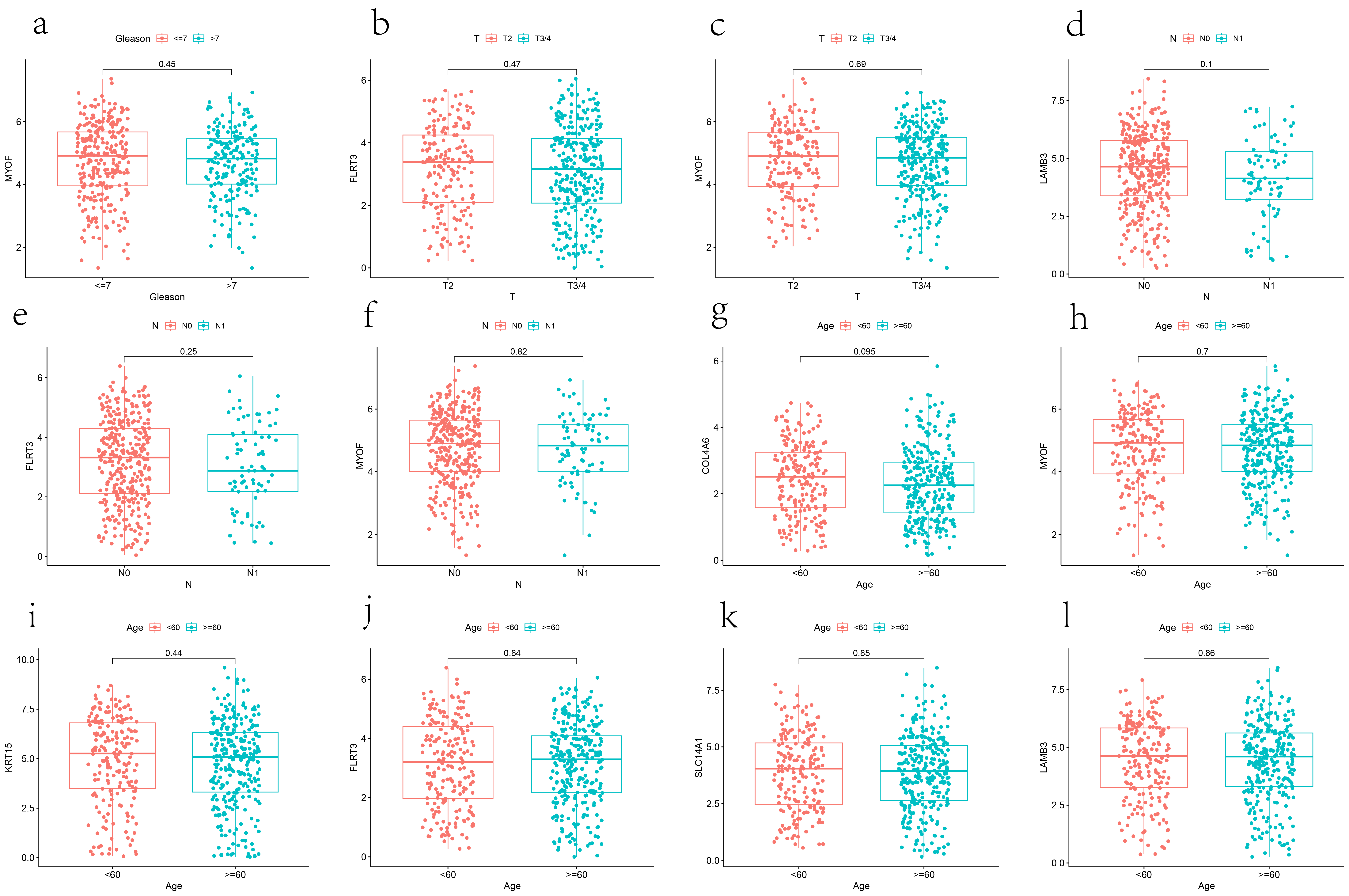


Supplementary Figure 2: Although the P value > 0.05, the expression of these 6 hub genes was relatively lower in patients with Gleason score >7, T3/4 stages, N1 stage, and age ≥60.

Supplementary Figure 3

Supplementary Figure 3: Immunohistochemistry data of SLC14A1, MYOF, KRT15, LAMB3, and FLRT3 from HPA database.

Supplementary Figure 4


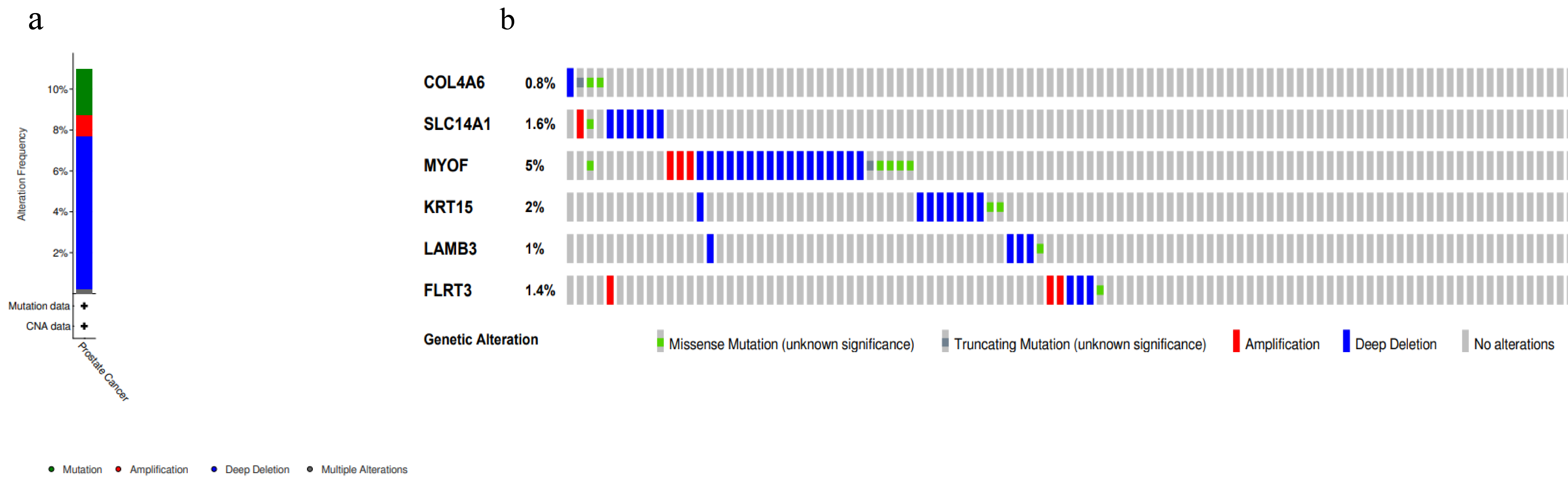


Supplementary Figure 4: Mutation and copy number alteration (CNA) analysis of 6 hub genes. (a) The mutations and CNA of 6 hub genes in prostate cancer patients. (b) The mutation and CAN of each gene.
